# Not All Conservatives Are Vaccine Hesitant: Examining the Influence of Misinformation Exposure, Political Ideology, and Flu Vaccine Acceptance on COVID-19 Vaccine Hesitancy

**DOI:** 10.3390/vaccines11030586

**Published:** 2023-03-03

**Authors:** Muhammad Ehab Rasul, Saifuddin Ahmed

**Affiliations:** 1 Department of Communication, University of California, Davis, CA 95616, USA; 2 Wee Kim Wee School of Communication and Information, Nanyang Technological University, Singapore 639798, Singapore

**Keywords:** COVID-19, vaccine hesitancy, flu vaccine, misinformation, public opinion, political ideology

## Abstract

Despite the mass availability of COVID-19 vaccines in the United States, many Americans are still reluctant to take a vaccine as an outcome from exposure to misinformation. Additionally, while scholars have paid attention to COVID-19 vaccine hesitancy, the influence of general vaccine hesitancy for important viruses such as the flu has largely been ignored. Using nationally representative data from Pew Research Center’s American Trends Panel survey (Wave 79), this study examined the relationship between perceived misinformation exposure, COVID-19 vaccine hesitancy, flu vaccine acceptance, political ideology, and demographic trends. The findings suggest that those who accepted the flu vaccine were less likely to be COVID-19 vaccine-hesitant. In addition, moderation analyses showed that perceived misinformation exposure increases COVID-19 vaccine hesitancy for conservatives and moderates but not for liberals. However, perceived misinformation exposure influences COVID-19 vaccine hesitancy among conservatives only if they are also flu vaccine-hesitant. Perceived misinformation exposure has no role in COVID-19 vaccine hesitancy if individuals (irrespective of political ideology) are regular with their flu vaccine. The results suggest that the effect of misinformation exposure on negative attitudes toward COVID-19 may be associated with generalized vaccine hesitancy (e.g., flu). The practical and theoretical implications are discussed.

## 1. Introduction

Since its emergence in 2019, the novel coronavirus, or SARS-CoV-2, has caused widespread destruction. While preventative measures such as mask wearing and social distancing have been effective ways to mitigate the adverse effects of COVID-19 [1], vaccine uptake is the long-term solution to control the virus and prevent further damage. However, despite the mass availability of vaccines in the United States, many people are hesitant to take vaccines. According to the Centers for Disease Control (CDC), only 69.1% of the population in the US is fully inoculated against COVID-19 [2]. Vaccine hesitancy can be characterized as the delay in acceptance, reluctance, or refusal to take a vaccine despite the availability of vaccination services [3]. Scholars have identified vaccine hesitancy as a significant challenge in the fight against COVID-19 [4,5]. Recent scholars have identified several factors that may contribute to this hesitancy, including the politicization of COVID-19 and apprehensions regarding the brisk development of the vaccines [6,7]. Particularly, researchers have pointed to the spread of and engagement with misinformation as a significant driver of COVID-19 vaccine hesitancy [8]. On the other hand, exposure to reliable information has been shown to increase the acceptance of vaccines [9].

The uncertainty and stress caused by COVID-19 resulted in an increased reliance on social media as individuals sought news about the pandemic and shared their opinions [10,11,12]. As such, social media platforms were inundated with information and emotional content, including uncivil discourse, especially regarding vaccines [13]. The overflow of mass information and emotional content sparked the genesis of an infodemic, which involved an unabated spread of information on social media, filled with misinformation and conspiracy theories about COVID-19 [14,15,16]. Existing research has argued that social media news is associated with greater engagement with COVID-19 misinformation [17]. In addition, the politicization of the virus across newspapers and network news likely contributed to misinformation related to COVID-19 [18]. Studies have found that right-leaning outlets referenced misinformation related to COVID-19 much more than left-leaning outlets [19]. The information ecology surrounding COVID-19 is inundated with false beliefs about the virus. When individuals are repeatedly exposed to misinformation, they are more likely to develop trust in those false beliefs through a phenomenon known as the illusory truth effect, which postulates that repeated information is seen as more credible than non-repeated information [20,21]. The consequences of the widespread misinformation are severe, as numerous studies have found evidence that exposure to COVID-19 misinformation has a detrimental effect on vaccine hesitancy [8,22,23]. As such, we posit our first hypothesis:

**Hypothesis** **1 (H1).** *Perceived misinformation exposure would be positively associated with COVID-19 vaccine hesitancy*.

Since its genesis, COVID-19 has been a heavily politicized issue, with Americans divided along partisan lines about the virus [24,25]. As an example, in 2020, only 18% of Democrats rated former President Donald Trump’s response to COVID-19 as good or excellent, while 83% of Republicans believed otherwise [26]. Using GPS data from mobile phones, one study found that Democrats were more likely to follow social distancing measures than Republicans [27]. Due to the politicization of COVID-19, political ideology has also impacted how people interact with misinformation about the virus and other issues. Indeed, studies have found that Republicans and conservatism are generally associated with increased susceptibility to and engagement with COVID-19 misinformation [28,29]. Further, consuming conservative media has been linked with increased susceptibility to COVID-19 misinformation [19]. In turn, consuming COVID-19 misinformation through political media sources could lead people to question the effectiveness of vaccines. For instance, one study found that people who believed in COVID-19 misinformation were less likely to get vaccinated due to skepticism about the vaccines’ swift development and low trust in the CDC and science [22]. Similarly, other studies have found that those who rely on social media for news are more likely to be vaccine hesitant due to skepticism about the vaccines, especially among those with lower levels of news literacy [8]. The politicization of the virus through media coverage is problematic. Researchers argue that it can sway public opinion because people rely on political actors to make decisions rather than science [30]. Additionally, politicized coverage can lead to increased polarization around an issue, resulting in motivated reasoning. People’s prior pro-attitudinal political attitudes drive information processing rather than new information, especially if it is counter-attitudinal [31]. In the context of COVID-19, people may be hesitant to take the vaccine due to their prior political beliefs, which will likely be reinforced as politicians fiercely debate the virus. In fact, researchers have found that Republicans hold negative attitudes about the COVID-19 vaccine [32], and have low intentions of getting vaccinated [33]. Further, studies have found that Republicans are more likely to believe that the COVID-19 vaccines have severe side effects and underestimate the scale of the clinical trials conducted to test the vaccines, resulting in a reluctance to take the COVID-19 vaccine [34]. It is important here to note that the partisan differences on the issue of the COVID-19 vaccines and the surrounding misinformation about the vaccines were parallel to false information about other polarizing topics, such as the 2020 election. Indeed, misinformation about the election and the COVID-19 vaccines was intertwined in public discourse leading up to the election [35]. As a result, conspiracy theories about the virus and the election spread unabated, largely from conservatives and right-leaning media sources, and were directed toward Democrats [19]. Existing research suggests that general perceived misinformation on vaccine hesitancy depends on political ideology. Hence, we propose the following hypothesis:

**Hypothesis** **2 (H2).** *Political ideology will moderate the relationship between perceived misinformation exposure and vaccine hesitancy such that the effects will be more substantial for conservatives than liberals*.

Due to the destructiveness of COVID-19, scholars and policymakers have focused on vaccine hesitancy related to the virus. However, seasonal flu, is another virus that spreads parallel to COVID-19 [36]. Medical researchers have established that the flu, combined with COVID-19, is highly detrimental to public health [37]. As a result, the CDC issued a strong statement urging people to vaccinate against the flu virus to avoid the risk of severe illness and death [38]. However, people may still be hesitant to vaccinate against the flu and COVID-19 due to misinformation about the vaccines. Since the start of COVID-19, the scholarly focus has been on political ideology and misinformation’s impact on COVID-19 vaccine hesitancy [8,19,23,27,28,29,32,33]. In this study, we also focus on flu vaccine acceptance to assess if COVID-19 vaccine hesitancy is specific to the pandemic or is related to general vaccine hesitancy. We argue that people who accept the flu vaccine should also accept the COVID-19 vaccine. Scholars have argued that the self-perceived risk of the flu and COVID-19 can impact the willingness to vaccinate [39]. One study found that people were more likely to get vaccinated against the flu due to the high perceived risk of COVID-19 at the start of the pandemic [40]. In the same study, people indicated a higher willingness to be vaccinated against COVID-19 and the flu if they had previously received a flu vaccine. These results suggest that intentions to vaccinate against the flu are related to vaccination intentions for COVID-19. Indeed, another study from Italy found that people were more likely to get vaccinated against COVID-19 and the flu at the start of the pandemic if they had received a flu vaccine before [41]. Hence, we pose the following hypothesis:

**Hypothesis** **3 (H3).** *Flu vaccine acceptance will be negatively associated with COVID-19 vaccine hesitancy*.

Recent research has also pointed out that political ideology may impact intentions to vaccinate against the flu. For example, one study found that Republicans were less likely to get vaccinated against the flu during COVID-19, with stronger Republicans’ showing a decreasing trend of intentions to vaccinate than Democrats [32]. Like the COVID-19 vaccine, perceived misinformation exposure related to myths about the flu can lead to vaccine hesitancy [42]. Researchers have argued that intentions to vaccinate against the flu may be higher if the perceived risk for COVID-19 is high [40]. However, it could be possible that intentions to vaccinate against the flu could impact the conditional role of political ideology on COVID-19 vaccine hesitancy. We argue that perceived misinformation exposure and political ideology may be related to flu vaccine acceptance, impacting COVID-19 vaccine hesitancy. We utilize existing flu vaccine attitudes to understand the effect of political ideology on COVID-19 vaccine hesitancy. Therefore, we pose the following research question:

**Research Question** **1 (RQ1).** *How does flu vaccine acceptance moderate the conditional relationship between perceived misinformation exposure, political ideology, and COVID-19 vaccine hesitancy*?

In summary, while significant advancements have been made in understanding COVID-19 vaccine hesitancy, existing gaps demand attention. First, while there are a few studies that examine flu vaccine hesitancy in conjunction with COVID-19 vaccine hesitancy [39,40,41], they primarily focus on the European context, while COVID-19 is much more politicized in the US [18]. Second, this body of research examines political ideology and flu vaccine acceptances as additional variables in some studies but fails to explore these relationships closely. Lastly, the vast majority of vaccine hesitancy research has rightfully focused on COVID-19 but has ignored flu vaccine hesitancy, which could play a critical role in understanding the characteristics of individuals who are reluctant to vaccinate against COVID-19 [40].

The current study addresses these gaps and explains how perceived misinformation impacts COVID-19 vaccine hesitancy. Specifically, we argue that perceived misinformation exposure leads to COVID-19 vaccine hesitancy dependent on political ideology. Then, we further extend the model and argue that these effects are further dependent (a moderated moderation) on acceptance of the flu vaccine (flu vaccine acceptance). Finally, identifying the characteristics of those likely to be vaccine hesitant can help scientists and policymakers further understand how to curb COVID-19 and reduce reluctance toward vaccination.

## 2. Materials and Methods

### 2.1. Data

We utilized data from the Pew Research Center’s (Washington, DC, USA) nationally representative American Trends Panel (ATP) Survey (Wave 79 = Pew 2020). This dataset was used as it contained the appropriate variables to test the relationship between perceived misinformation exposure, vaccine hesitancy, flu vaccine acceptance, and political ideology. Furthermore, other studies have relied on ATP data to draw conclusions about COVID-19 [19]. The ATP survey was conducted between 19 November and the 28 November 2020 (*n* = 12, 648) (see “The American Trends Panel Survey Methodology” for more details). The data and analyses are weighted. The dataset is publicly available on the Pew Research Center’s (Washington, DC, USA) website upon request.

### 2.2. Measures

*COVID-19 vaccine hesitancy (dependent variable)* was measured by asking participants if they would get the COVID-19 vaccine if it were made available today. The responses ranged from 1 = definitely get the vaccine to 4 = definitely not get the vaccine (*mean* = 2.29; *SD* = 1.07).

*Perceived misinformation exposure (independent variable)* was measured through a single-item that asked participants to report whether they had encountered information or news related to the 2020 US presidential election that seemed to be fabricated. This item was reverse coded. The recoded responses ranged from 1 = none at all to 4 = a lot (*mean* = 2.95; *SD* = 0.86). We follow the approach of existing studies which use self-reported items to measure perceived misinformation exposure [43,44,45].

*Political ideology (moderator)* was measured through a single item asking participants to indicate their political ideology. This item was reverse coded. The responses ranged from 1 = very liberal to 5 = very conservative (*mean* = 3.08; *SD* = 1.05).

*Flu vaccine acceptance (moderator)* was assessed through two items asking participants to indicate whether they had received a flu shot since August 2020 (are currently vaccinated, 1 = no, 2 = yes) and how frequently they get the flu shot (1 = rarely, 2 = every few years and 3 = every year). The items were multiplied to create a flu vaccine acceptance score allowing us to create three categories of flu vaccine getters (scores of 1–3 = hesitant, 4 = irregular, and 6 = regular). The hesitant group includes individuals who did not recently take a flu vaccine or those who took a flu vaccine but rarely take it. The irregular group includes flu-vaccinated individuals, but they usually take it only every few years. The regular group includes currently flu-vaccinated individuals who take it every year (*mean* = 3.29; *SD* = 2.23).

### 2.3. Covariates

Several demographic variables were included as covariates as they may influence vaccine hesitancy. These variables include (a) religion (42.2% Catholic, 20.9% Protestant, 27.7%, unaffiliated, 9.2% others), (b) age (1 [18–29 years], 2 [30–49 years], 3 [50–64 years], 4 [65+ years]; *mean* = 2.48, *SD* = 1.02), (c) education (1 [less than high school] to 6 [postgraduate]; *mean* = 3.43, *SD* = 1.60, *median* = 3 [some college, no degree]), (d) gender (52% female), (e) marital status (51.7% married), (f) income (1 [less than $30,000] to 9 [$100,000 or more]; *mean* = 3.43, *SD* = 1.60, *median* = 4 [$50,000 to less than $60,000]), and (g) race (73.4% White, 12.3% Black, 5.4% Asian, 4% mixed, 4.9% other).

### 2.4. Analysis

We employed ordinary least squares (OLS) regression to examine the impact of perceived misinformation exposure and flu vaccine acceptance on vaccine hesitancy. In addition, we employed a two-way and three-way interaction moderation analysis using SPSS PROCESS macro v4.2 [46], to assess the moderating role of political ideology and flu on the relationship between perceived misinformation exposure and vaccine acceptance.

## 3. Results

First, we ran regression analyses to predict COVID-19 vaccine hesitancy. The results suggested that those who accepted the flu vaccine (*β* = −0.347, *p* < 0.001) were less likely to be COVID-19 vaccine-hesitant. We found that those who perceived to be exposed to misinformation were more likely to be vaccine hesitant (*β* = 0.019, *p* <.05).

Additionally, we observed that those who identified as female (*β* = 0.157, *p* < 0.001), black (*β* = 0.087, *p* < 0.001), married (*β* = 0.033, *p* < 0.001), and low-income individuals (*β* = −0.032, *p* = 0.001) were more likely to be COVID-19 vaccine-hesitant (see Table 1 for additional details).

On the other hand, we found that those who identified as Asian (*β* = −0.056, *p* < 0.001), individuals belonging to other racial groups (*β* = −0.026, *p* < 0.01), protestant (*β* = −0.082, *p* < 0.001), unaffiliated with a religion (*β* = −0.044, *p* < 0.001), those belonging to other religions (*β* = −0.056, *p* < 0.001), individuals between 50–64 years old (*β* = −0.075, *p* < 0.001), and individuals over 65 years old (*β* = −0.142, *p* < 0.001) were less likely to be COVID-19 vaccine hesitant.

Next, to examine the process through which political ideology and flu vaccine acceptance impact the relationship between perceived misinformation exposure and vaccine hesitancy, we ran moderation analyses (Model 1 and Model 3) with perceived misinformation exposure as the predictor variable, political ideology, and flu vaccine acceptance as moderators, and vaccine hesitancy as the outcome variable using SPSS PROCESS macro v4.2 [46]. The bootstrapping method was used to approximate the conditional effects (*n* = 5000).

**Figure 1:**
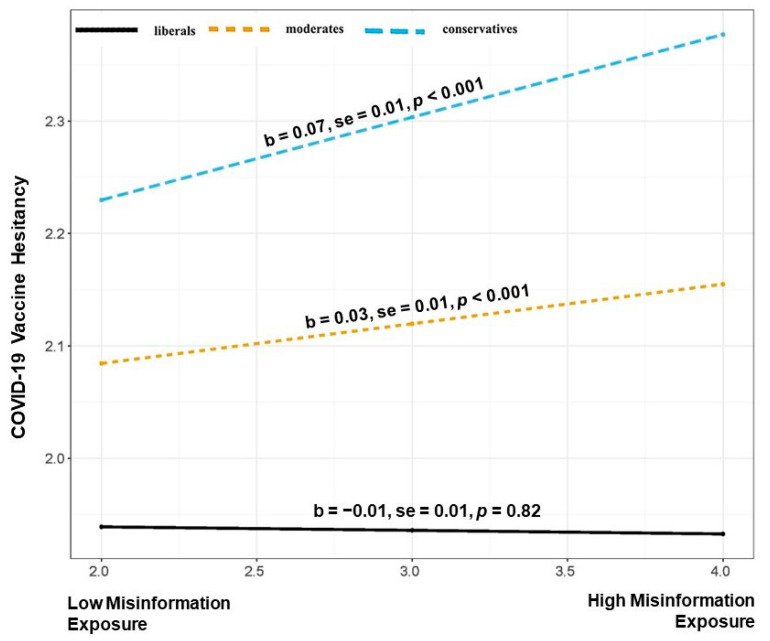
Illustration of the interaction between perceived misinformation exposure and political ideology in predicting COVID-19 vaccine hesitancy.

First, we ran a two-way interaction (Model 1) using political ideology, perceived misinformation exposure, and vaccine hesitancy. The formal statistical test of the moderation process revealed that the conditional effects were statistically significant (B = 0.038, SE = 0.009, *p* < 0.001). The relationship is illustrated in Figure 1. We find that an increase in perceived misinformation exposure is associated with COVID-19 vaccine hesitancy only among moderates (b = 0.03, SE = 0.01, *p* < 0.001) and conservatives (b = 0.07, SE = 0.01, *p* < 0. 001) but not liberals (b = −0.01, SE = 0.01, *p* = 0.82).

**Figure 2:**
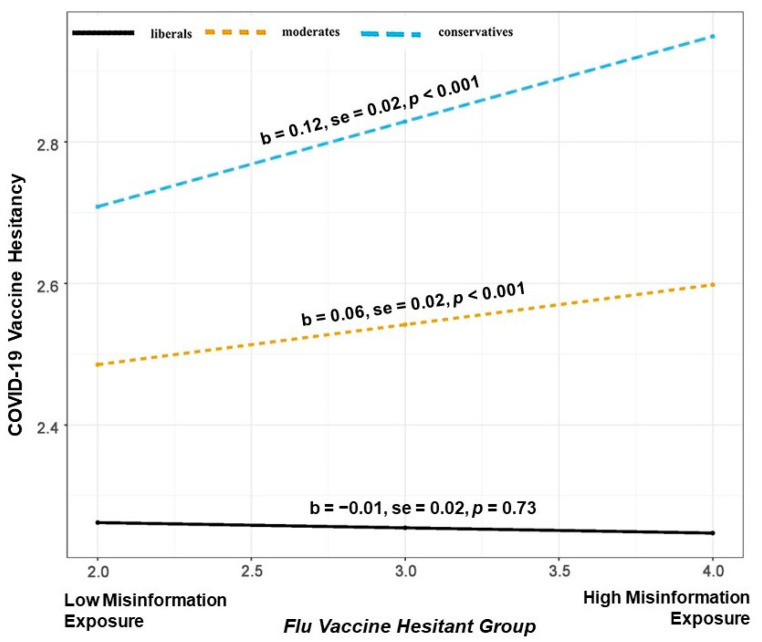
Illustration of the interaction between perceived misinformation exposure and political ideology in predicting COVID-19 vaccine hesitancy for the flu vaccine hesitant group.

**Figure 3:**
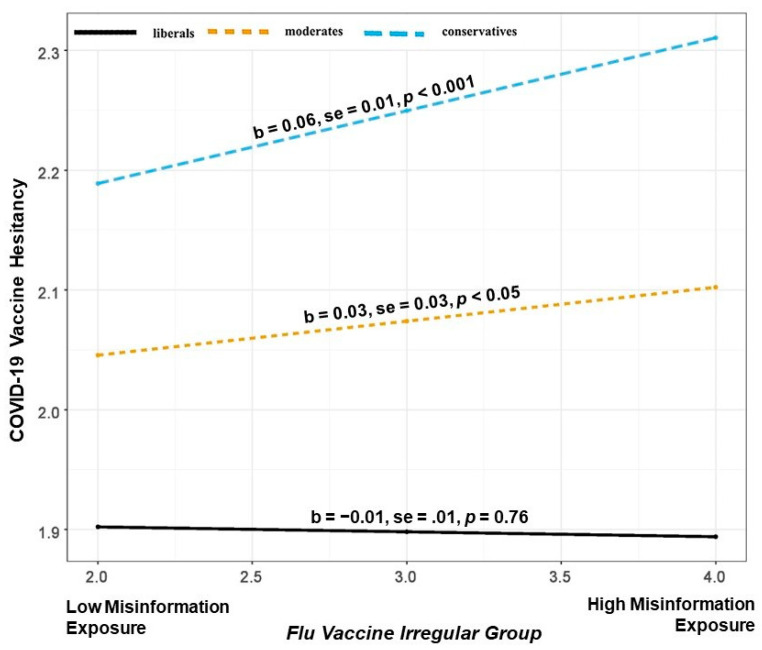
Illustration of the interaction between perceived misinformation exposure and political ideology in predicting COVID-19 vaccine hesitancy for the flu vaccine irregular group.

**Figure 4:**
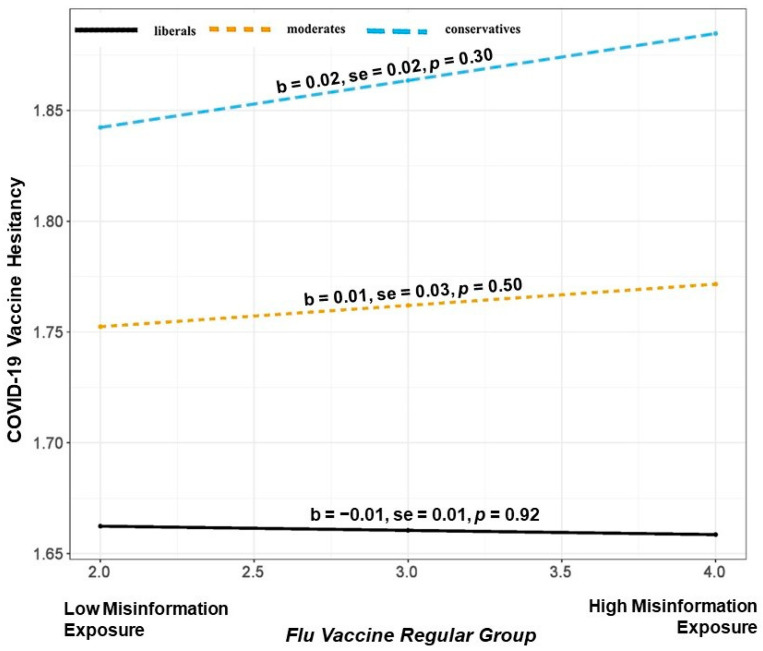
Illustration of the interaction between perceived misinformation exposure and political ideology in predicting COVID-19 vaccine hesitancy for the flu vaccine regular group.

Next, we ran a three-way interaction (Model 3) between political ideology, flu vaccine acceptance, perceived misinformation exposure, and vaccine hesitancy. The moderated moderation was statistically significant (b = −0.011, SE = 0.004, *p* < 0.001). This suggests that the moderation of perceived misinformation exposure through political ideology on vaccine hesitancy depends on the levels of flu vaccine acceptance. The relationship is plotted in Figure 2, Figure 3 and Figure 4.

Probing the interaction suggests that the conditional relationship between perceived misinformation exposure and political ideology in predicting COVID-19 vaccine hesitancy is significant only for those who are also flu vaccine-hesitant (those individuals who are not flu vaccinated or those who rarely take it, Figure 2) and irregulars (currently flu-vaccinated individuals but they usually take it only every few years, Figure 3). However, the conditional relationship is not significant for those who regularly use their flu vaccines (currently flu-vaccinated individuals who take flu vaccine every year (Figure 4).

As illustrated in Figure 2, among the flu vaccine hesitant, an increase in perceived misinformation exposure is associated with COVID-19 vaccine hesitancy among conservatives (b = 0.12, SE = 0.02, *p* < 0.001) and moderates (b = 0.06, SE = 0.02, *p* < 0.001). Alternatively, as seen in Figure 3, we observe weakened effects among flu vaccine irregulars. Here, an increase in perceived misinformation exposure is associated with COVID-19 vaccine hesitancy among conservatives (b = 0.06, SE = 0.01, *p* < 0.001) and moderates (b = 0.03, SE = 0.03, *p* < 0.05). Perceived misinformation exposure does not impact COVID-19 vaccine hesitancy for either conservatives, moderates, or liberals if they are regular with their flu vaccine (see Figure 4).

## 4. Discussion

Vaccine hesitancy is a critical issue not just during COVID-19 but also for other diseases, such as the flu. The politicization of vaccines has severe implications for populations worldwide. While numerous studies have examined the relationship between misinformation, political ideology, and COVID-19 vaccine attitudes [8,18,19,22,23,27,28,29], very few have looked at other important factors, such as general vaccine attitudes [39,40,41]. Importantly, the existing literature has not considered the factors mentioned above in conjunction. The current study aimed to bridge this gap by examining the relationship between perceived misinformation exposure and COVID-19 vaccine hesitancy by paying attention to both political ideology and flu vaccine acceptance.

The findings of this study provide an essential insight into the impact of misinformation on vaccine hesitancy for COVID-19 and the flu, especially in the US, where misinformation, especially about COVID-19 and public health, is prevalent online and offline [18,19]. Moreover, existing scholarship has overwhelmingly focused on the COVID-19 vaccine, ignoring the vital role of the flu vaccine. Reluctance to get vaccinated and the politicization of vaccines are issues that have been present for decades. Thus, this study contributes meaningfully to the existing literature by reestablishing the flu vaccine, among other variables, as an essential factor in understanding COVID-19 vaccine hesitancy.

The results suggest those with high levels of flu vaccine acceptance were less likely to be hesitant to take the COVID-19 vaccine. Additionally, perceived misinformation exposure was positively related to COVID-19 vaccine hesitancy. Furthermore, the two-way moderation analyses revealed that people who identify as moderate and conservative are more likely to be reluctant to take the COVID-19 vaccine if they perceive to have been exposed to misinformation. The three-way moderation analyses revealed that conservatives are more likely to be COVID-19 vaccine hesitant if they are also hesitant and irregular with flu vaccines. Lastly, no COVID-19 vaccine hesitancy is found, irrespective of political ideology, for those who are regular with their flu vaccines. In addition to these results, we also ran additional analyses with political ideology as a condensed variable (see Supplementary Materials for more detail).

The genesis of an infodemic during COVID-19 has resulted in the mass availability and unabated spread of misinformation online and offline. A large body of research has found that misinformation can lead to vaccine hesitancy in the context of COVID-19 [8,22,23,47]. Therefore, the results suggesting that perceived exposure to misinformation is directly associated with COVID-19 vaccine hesitancy are not surprising. However, scholars should continue to focus on minimizing the impact of misinformation on general vaccine hesitancy in addition to COVID-19 vaccine hesitancy. Although policymakers and public health officials have developed programs to educate people about the effectiveness of the COVID-19 vaccines, recent studies offer additional countermeasures that can curb the impact of misinformation on vaccine hesitancy. For example, some scholars have found news literacy to serve as a buffer against COVID-19 [8], while others have argued that focusing on health literacy and minimizing negative emotion in COVID-19 messaging helps fight against misinformation [48].

It is important to note that despite the overwhelming scholarly focus on COVID-19 vaccine hesitancy, it is not a novel issue. For instance, one study found that 43% of Americans believe in misperceptions about the flu vaccine and are reluctant to take the vaccine despite corrective interventions [42]. Moreover, scholars have found that people vaccinated against the flu are more likely to get vaccinated against COVID-19 due to increased perceived risk of the virus [40]. Given the flu vaccine’s association with the COVID-19 vaccine, it is likely that acceptance of the flu vaccine explains why it is negatively associated with reluctance to uptake the COVID-19 vaccine.

The findings from our study highlight that the relationship between perceived misinformation exposure and COVID-19 vaccine hesitancy is dependent on political ideology. We also find that acceptance of the flu vaccine is a critical factor in this relationship. Specifically, conservative individuals are more likely to be reluctant to take COVID-19 if they are also hesitant about the flu vaccine. This suggests that COVID-19 vaccine hesitancy may not be specific to COVID-19. The conservatives who are regular with their flu vaccines are not found to be COVID-19 vaccine-hesitant.

Overall, these results corroborate existing research highlighting the highly politicized nature of COVID-19, as exemplified by the partisan coverage of the virus where right-leaning news outlets amplified the spread of misinformation about the virus [19]. Furthermore, studies have found that conservatives are less likely to get vaccinated against COVID-19 [32,33]. The findings also support the partisan-motivated reasoning framework [49], which suggests that people prioritize information that aims to protect their partisan identity. In turn, they miss out on relevant information that can assist in making important decisions [50], such as taking the COVID-19 vaccine.

It is important to place this finding in the right context. The data used in this study were collected right after the 2020 US election. In addition, the measure assessing perceived misinformation in the current study focused on the election rather than the COVID-19 vaccine. Republicans and conservative commentators have referred to COVID-19 as a “hoax,” questioned the science underlying the vaccine [19] and used it as a rallying cry in the lead up to the 2020 US election. Moreover, Donald Trump, the US president at the time, led the Republican party’s attacks on the news and undermined the credibility of news organizations. Indeed, researchers have found that support for Trump is positively related to increased distrust in the news and media [51]. There is also evidence that people who distrust the media do not rely on mainstream sources but rather focus on alternative sources [52], such as social media [53]. In turn, these alternate sources may fuel perceptions of misinformation [54]. As such, it is unsurprising that even perceived misinformation exposure not directly related to COVID-19 is associated with conservatives’ hesitancy towards vaccines. This, combined with the politicized coverage of the pandemic [18], explains why political ideology is a significant determinant of whether people will get vaccinated against COVID-19.

The current study has practical and theoretical implications for public health practitioners, policymakers, and scholars. First, over the last couple of years, the overwhelming scholarly focus has been rightly on vaccine hesitancy related to COVID-19. However, vaccine hesitancy is not a novel issue. It is related to other important vaccine hesitancy issues, such as the flu and human papilloma virus (HPV) vaccine [55]. As such, scholarship and policies must also focus on general vaccine hesitancy rather than on a specific vaccine.

Further, while numerous studies have pointed out the importance of political ideology as an important factor in misinformation belief and vaccine hesitancy in the context of COVID-19 and beyond [18,19,28,29,32,33]. However, very few interventions have been developed with political ideology as a focus. Accordingly, more work is needed to counter the effect of political ideology on decision-making in a health context. Theoretically, the current study potentially shows the application of the motivated reasoning and partisan motivated reasoning frameworks in a health context [31,49]. As a matter of fact, scholars have begun to examine COVID-19 vaccine hesitancy and misinformation through the partisan-motivated reasoning framework [56]. Nonetheless, as with any academic inquiry, more work is needed to understand how to counter the harmful effects of partisan-motivated reasoning.

Lastly, the current study is not without limitations. While utilizing the ATP data from Pew allows us to use a demographically rich and large sample, we cannot reach any causal inferences due to the cross-sectional nature of the data. The findings of the study are also limited to one context where COVID-19 has been highly politicized and, therefore, may not translate into other contexts. In addition, the measure for perceived misinformation exposure is measured by one item, which limits robust conclusions about vaccine hesitancy. Researchers have argued that single-item measures lack internal consistency and often do not capture the complex nature of psychological constructs [57]. Multiple items allow for a more nuanced understanding of a construct [58]. In the context of the current study, we were limited by the measure used by Pew in the ATP survey and urge readers to interpret our results cautiously. Ultimately, we focused on political ideology and acceptance of the flu vaccine as two factors that impact the relationship between perceived misinformation exposure and COVID-19 vaccine hesitancy. However, there may be additional political variables that may impact this relationship as well.

## 5. Conclusions

Despite the development of vaccines, COVID-19 continues to plague the world. However, people continue to be exposed to misinformation about the virus and resist COVID-19 vaccines. This leaves certain groups vulnerable to the hazardous short-term and long-term effects of COVID-19. We urge academics and policymakers to consider this study’s findings when designing interventions to minimize the harmful effects of misinformation on vaccine hesitancy. For instance, messages and interventions focused on reducing vaccine hesitancy should highlight the importance of the flu vaccine in conjunction with the COVID-19 vaccine. Additionally, efforts should be made to correct commonly held misperceptions about vaccines held by moderates or conservatives. Recent studies have found some success in correcting health misinformation through news literacy interventions [59]. However, interventions focused on the flu and COVID-19 vaccines in combination with correcting vaccine-related misinformation are scarce. As such, we hope that this study can assist academics and policymakers in developing interventions that are effective in reducing vaccine hesitancy.

## Figures and Tables

**Table 1:** Predicting COVID-19 vaccine hesitancy.

Variable	COVID-19 Vaccine Hesitancy	
	*Β*	Std. Error
Protestant	−0.082 ***	0.024
Unaffiliated	−0.044 ***	0.023
Others	−0.056 ***	0.033
30–49 Years Old	−0.004	0.026
50–64 Years Old	−0.075 ***	0.028
65+ Years Old	−0.142 ***	0.031
Education	−0.006	0.006
Income	−0.032 ***	0.003
Female	0.157 ***	0.018
Others	0.013	0.090
Marital Status	0.033 ***	0.020
Black	0.087 ***	0.029
Asian	−0.056 ***	0.039
Mixed Race	0.011	0.045
Other Race	−0.026 **	0.042
Political Ideology	0.165 ***	0.009
Flu Vaccine Acceptance	−0.347 ***	0.004
Perceived Misinformation Exposure	0.019 *	0.010
**Total R^2^**	0.252	

Statistical significance is marked as * *p* < 0.05; ** *p* < 0.01; *** *p* < 0.001.

## Data Availability

The data for this study is publicly available upon request at https://www.pewresearch.org/science/dataset/american-trends-panel-wave-79/ (accessed on 7 January 2023).

## Supplementary Materials

The following supporting information can be downloaded at: https://www.mdpi.com/article/10.3390/vaccines11030586/s1, Table S1. Predicting COVID-19 vaccine hesitancy.
