# Supplementary material for: Not All Conservatives Are Vaccine Hesitant: Examining the Influence of Misinformation Exposure, Political Ideology, and Flu Vaccine Acceptance on COVID-19 Vaccine Hesitancy
Source: Vaccines (Basel). 2023 Mar 3;11(3):586. doi: 10.3390/vaccines11030586 (PMC10052187; doi:10.3390/vaccines11030586)
Supplement: Supplementary file 1 [file vaccines-11-00586-s001.zip › vaccines-2216147-supplementary.pdf]

Supplementary Materials

Table S1. Predicting COVID-19 vaccine hesitancy

Variable	COVID-19 Vaccine Hesitancy	
	β	Std. Error
Protestant	-0.081***	0.024
Unaffiliated	-0.048***	0.023
Others	-0.057***	0.033
30-49 Years Old	-0.001	0.026
50-64 Years Old	-0.074***	0.028
65+ Years Old	-0.139***	0.031
Education	-0.010	0.006
Income	-0.033***	0.003
Female	0.158***	0.018
Others	0.008	0.090
Marital Status	0.035***	0.020
Black	0.090***	0.029
Asian	-0.054***	0.040
Mixed Race	0.012	0.045
Other Race	-0.025**	0.042
Political Ideology	0.152***	0.008
Flu Vaccine Acceptance	-0.349***	0.004
Perceived Misinformation Exposure	0.019*	0.011
**Total R^2**	0.252	

[a]*Statistical significance is marked as *p < 0.05; **p < 0.01; p < 0.001***.*

[b]*Political ideology is treated as a condensed variable here (1-3; liberal, moderate, and conservatives)*
